# Evaluation of the effects of comprehensive reform on primary healthcare institutions in Anhui Province

**DOI:** 10.1186/1472-6963-14-268

**Published:** 2014-06-19

**Authors:** Qing Liu, Xin Tian, Jiang Tian, Xinping Zhang

**Affiliations:** 1 Tongji Medical College, Huazhong University of Science and Technology, Hubei Province, China; 2 Social Insurance Administration, Ministry of Human Resources and Social Security, Beijing, China; 3 Beijing Friendship Hospital, Capital Medical University, Beijing, China; 4 Health Human Resources Development Center, Ministry of Health, Beijing, China

**Keywords:** Health reform, Primary healthcare institutions, Anhui province

## Abstract

**Background:**

In 2009, the Chinese Central Communist Party and the China State Council started to implement comprehensive healthcare reforms. The first round of reforms, involving Anhui province, was from 2009 to 2011, and focused on primary healthcare institutions. This study conducts an initial assessment of the effects of specific parts of the reforms in Anhui.

**Methods:**

Mixed quantitative and qualitative methods were adopted for data collection. Seven hundred and three health institutions from 15 counties were randomly chosen. The practices, development, effects, problems, and other relevant information related to the reform were classified into four aspects: medicine management; personnel systems and income distribution mechanisms; compensation mechanisms for primary healthcare institutions; and strengthening the primary healthcare system. The effects of reform were analyzed by evaluating changes in compensation channels, visit costs, diagnosis and treatment structure, hardware, structures, efficiency, and behavior.

**Results:**

A new system for authorizing drugs resulted in a total of 857 new drugs being accessible at agreed prices through primary healthcare institutions in Anhui. The cost of the average outpatient visit decreased from 35.29 RMB to 31.64 RMB, although for inpatients, the average cost increased from 799.05 RMB to 992.60 RMB. The number of healthcare personnel decreased, but their workloads increased. The total revenue from government sources increased by 41.09%, and the proportion of revenue from drugs decreased by 25.19%. The rate of diagnosis and treatment visits and outpatient visits to primary healthcare institutions increased. Finally, between 2008 and 2010, 1,195 standardized township hospitals, 14,134 village clinics, and 1,234 community health service institutions were constructed.

**Conclusion:**

The reform of primary healthcare institutions in Anhui has improved the personnel structures surrounding frontline healthcare workers, increased their incomes, improved work efficiency, and changed the compensation patterns of primary healthcare institutions, improved hardware, reduced drug prices, and, to some extent, improved the diagnosis and treatment structure. However, the reforms have not radically changed the behavior of medical workers or the visit patterns of patients. Approaches such as strengthening performance evaluation, and carrying out initiatives to further mobilize frontline healthcare workers, enhance rational drug use through improved training and educate patients, should be undertaken in the future.

## Background

Since China’s reform and opening up policy, its medical burden has increased more rapidly than its GDP growth. But a lot of problem existed in the primary health institutions in China. Before the 2007 reform, the average cost of an inpatient visit to a primary healthcare institution was 2,497.10 RMB, equivalent to 60.3% of the rural per capita income. Unfortunately, however, the fairness of China’s health services has declined sharply. Ranked by fund-raising and fairness of distribution of healthcare, China was the antepenultimate country of 191 in the World Health Report 2000. The root of the problem might be that the direct government subsidy for public health institutions in China is relatively small, forcing these institutions to rely on drug profits for additional income [1]. Therefore, a number of serious problems emerged due to stimulated overtreatment and irrational drug use [2-5]. Before the 2007 reform, government financial compensation accounted for 21.6% of the total income of urban community health service centers, and 24.2% of that of rural township hospitals. By contrast, drug revenue accounted for 50.2% of the total income of urban community health institutions, and 39% of that of rural township hospitals [6]. Another attention was paid on the level of medical staff of primary healthcare institutions. Large numbers of medical personnel without formal medical education were employed, so the staff of primary healthcare institutions generally had widely different levels of professional qualifications [7].

In 2009, the Chinese Central Communist Party and the China State Council started to implement a comprehensive healthcare reform initiative called “Opinions on Deepening Pharmaceutical and Healthcare System Reform” to improve the current status in the primary healthcare institutions. The central government was responsible for the overall policy design and increased investment in the local healthcare system. Local governments were responsible for implementation of the reform policy. The whole reform consisted of three rounds and the target of the whole reform was to finally solve the problems in the primary healthcare institution in China by returning the public welfare nature of primary care institutions, reducing patients’ burden of medical expenditure, improving the quality of services and improve the quality and motivation of the healthcare staff. During the first round of the reform, the total national financial health expenditure was 1.5166 trillion RMB, of which 450.6 billion RMB came from central government. This was an additional 1.2409 trillion RMB over those 3 years, compared with 2008. The additional investment was mainly used for five aspects: constructing the basic medical insurance system, establishing the national essential drug system, improving the system of primary healthcare services, improving to a similar basic level all public health services, and conducting the pilot reform of public hospitals. Primary healthcare institutions were key to the whole healthcare system. Strengthening the capacity of primary care could relieve pressure on hospitals, inhibit the rapid increase in medical costs, and increase equity of access to health services.

Anhui province was one of the several provinces involved in the first round of the reforms, and the reforms in this province covered four areas: 1) implementation of the essential drug system and drug procurement mechanism, 2) improving personnel structures, 3) establishment of the new compensation mechanism for institutions and 4) strengthening the primary healthcare system by building new hospitals and clinics [8]. The primary healthcare institutions included both urban and rural institutions [8]. This study summarizes the way in which the reforms were carried out in rural healthcare institutions in Anhui. The initial assessment of the effects may provide lessons for implementing the reforms elsewhere in China or in other developing countries.

## Methods

### Settings

Anhui is located in eastern China, with a total area of 139,600 km^2^ and a population of 61.18 million in 2010. The GDP of Anhui reached 1.22634 trillion RMB in 2010, 14^th^ among the 31 provinces in China, including municipalities and autonomous regions. Its GDP per capita was 16,656 RMB, 17^th^ in China [9]. There are four regions, 12 larger cities, 10 smaller cities and 56 counties in Anhui province. Thirty-two counties were involved in the first round of reforms, 15 of which were chosen at random for inclusion in our study. There are 3,363 rural healthcare institutions throughout the province, of which 1,571 are urban community health service institutions and 1,792 rural township health institutions. We chose 703 rural health institutions at random for this study. The counties chosen were Luyang district, Wuhu county, Guzhen county, Fengtai county, Zhixi county, Shizishan district, Tongling county, Yuexi county, He county, Langya district, Yingshang county, Yongqiao county, Tangshan county, Juchao district, and Guizhi district.

### Data collection

The study used a combination of quantitative and qualitative methodology to collect data on the effects of the reforms.

First, we carried out a review of the literature including policy documents, reports, academic articles, and interview and seminar materials. Policy documents relating to the reform of primary healthcare institutions and dating from the start of reform in August 2009 were collected from the medical reforms website and Anhui’s health bureau website. EMBASE, PubMed, WHO, INRUD, Google, and other foreign databases and websites, as well as CNKI, Chongqing VIP, Wanfang, and other Chinese periodical websites were used to search relevant academic publications and research reports from 2010.

Second, this study collected information using structured face-to-face interviews and seminars. The interviewees consisted of three government policymakers, three administrators of primary healthcare institutions implementing the reforms, three frontline medical workers from primary healthcare institutions implementing the reforms, and one individual in charge of government drug procurement. The main interview questions for policymakers covered the process of policymaking, problems related to policy implementation, and their ideas and suggestions for policy adjustment. The main interview questions for administrators covered the functioning of and major changes to primary healthcare institutions, implementation of relevant policy, reform-related problems, and suggestions for changes to the reforms. The main interview questions for frontline healthcare workers covered the changes in health service behaviors, income, work efficiency and satisfaction, and their suggestions for changes to the reforms. The main interview questions for the individual in charge of government drug procurement covered the specific systems for procurement, results of bidding, and problems encountered in the bidding process and procurement of essential drugs. All information was obtained with the consent of the interviewees. We held three seminars in January, June, and December 2010, inviting two reform policymakers, two experts and scholars on health management and health economics, and two primary healthcare administrators to discuss the current status of reforms, the effects so far, emerging problems, and ideas for changes to the policy. Specific questionnaire was designed for the interviews and seminars. To ensure the quality of investigation, the unit chief and the people who filled in the questionnaire were asked to thoroughly check the questionnaire and affix their names.

Thirdly, the prescription survey used random sampling and concealed both patient and doctor information. Data for the prescription survey were collected on the first Mondays of March, July, and November 2009 and 2010; that is, 3 days a year and 6 days in total. Twenty prescriptions were randomly drawn from each institution on each of those 6 days, giving a total of 120 prescriptions for each institution. The drug information in each was entered into the database. The antibiotics were identified and the proportion of prescriptions including antibiotics was calculated. This work was done by investigators employed by the research group.

All the data in this study were collected through site investigations of primary healthcare institutions, from annual statistical reports of these institutions and samples of prescriptions. The statistical reports chosen were largely those that had been reported to the county health administrative departments and only made public after being checked.

### Data analysis

The issues emerging from the reforms were classified into four areas: medicine management, the personnel system and income distribution mechanism, the compensation mechanisms for institutions, and strengthening the primary healthcare system.

Indicators for evaluating the effects on medicine management in primary healthcare institutions to reduce the patients’ burden of medical expenditure and return the public welfare nature of primary care institutions, included (1) number of named drugs gaining single supplier status; (2) average cost of an outpatient visit, or total outpatient revenue/total outpatient visits; (3) average cost of an inpatient visit, or total inpatient revenue/total inpatient visits.

Indicators for evaluating the effects on personnel systems and income distribution mechanisms in primary healthcare institutions to improve the quality and motivation of the healthcare staff, included (1) proportion of qualified to unqualified staff; (2) frontline healthcare workers’ average workload, and (3) annual diagnosis and treatment visits/total number of health staff, whether qualified or unqualified.

Indicators for evaluate the effects on the compensation mechanisms for primary healthcare institutions to reduce the patients’ burden of medical expenditure and return the public welfare nature of primary care institutions, included (1) number of outpatient visits; (2) proportion of total institutional income deriving from drugs and medicines; (3) number of inpatient visits; (4) annual change in outpatient visits; (5) annual change in discharge rates; (6) proportion of total institutional income derived from government financial aid, and (7) proportion of prescriptions for antibiotics in all the prescriptions.

Indicators for evaluating whether or not the primary healthcare system has been strengthened to improve the quality of the primary healthcare institutions included number of new healthcare institutions.

The research group members completed written records, post-interview settlement, and summaries with the interviews and seminars.

### Ethics statement

The study did not involve any ethical issues. Investigations were permitted by every primary healthcare institution, and data obtained were approved for use for academic research purposes. All information was obtained with the consent of the interviewees and those who attended the seminars.

## Results

The main effects of primary healthcare reform in Anhui were as follows:

1. **Medicine management after the comprehensive reform**

Before the reforms, primary healthcare institutions could only be supplied with medicines deemed essential, which consisted of 307 drugs in the national essential drugs list and 255 in the Anhui supplementary drugs list. No other medicines were authorized for use in these institutions.

The revised bidding and procurement process for essential medicines ran as follows, summarized by the government policymakers and administrators of primary health care institutions implementing reform:

1) Medicine manufacturers bid for the opportunity to supply medicines from the essential lists and were responsible for the continuing supply of drugs;

2) The bidding and procurement processes were unified into a single step, led by the provincial drug bidding and procurement center;

3) The “double envelope” method was adopted, where both technical and business standards had to be met. Drugs and manufacturers were required to meet the technical standards before they were eligible to bid. All the drugs and manufacturers were ranked and scored for a range of indicators, including the corporate Good Manufacturing Practice certification, quality, type, scale of production, sales, industry rankings, market reputation, and adverse record. If they met a certain minimum standard, then the manufacturers underwent the business standard review to offer prices. Those who offered the lowest prices won the contract;

4) Each essential medicine was obtained from a single source. In principle, there should be only one supplier for each essential medicine to ensure that they are guaranteed the province’s custom.

In September 2010, Anhui completed the centralization of the bidding and procurement system for essential medicines. A total of 9,676 drugs from 1,202 manufacturers all over the country participated in the process. There were 7,616 drugs that met the technical standards, and of these, 4,422 met the business standards. In other words, 42% of technically suitable drugs failed to meet the business standards. At the end of the process, 857 products had been procured to the necessary technical standards, and at the lowest possible prices. The overall quality of medicines improved, with 34.6% of drugs now coming from the top 100 medical enterprises, and 57.6% of drugs coming from the top 400 medical enterprises. Largely as a result of the changes to drug costs, the cost of the average outpatient visit to primary healthcare institutions declined continuously from 2008 to 2010 (Table 1).

**Table 1:** Changes in inpatient and outpatient visit costs in primary healthcare institutions

	**2008**	**2009**	**2010**
Average outpatient visit cost in primary health care institutions (RMB)	35.29	32.70	31.64
Average inpatient visit cost in primary health care institutions (RMB)	799.05	747.03	992.60

Data were summarized from the statistical reports chosen.

Before the implementation of the reforms, the proportion of prescriptions that included antibiotics was 65.57%. After the reforms, that fell to 64.95%, or a reduction of 0.62%, which is not statistically significant. This suggests that the prescribing behavior of healthcare professionals did not change as a result of the reforms.

2. **Personnel systems and income distribution mechanisms after the comprehensive reform**

After the reforms, evaluation focused on the number of services, quality, effectiveness and resident satisfaction, and information about government compensation and medical staff earnings.

The staff of Anhui’s public primary healthcare institutions all competed to be rehired, taking a written examination and undergoing an interview. Staff who failed in either were given several options. The first option was early retirement. The second was transition resettlement, in which individuals were given a 3-year cushioning period for further study. Afterwards, they could reapply for jobs and be prioritized for re-hiring under the same conditions. The third option was to encourage self-employment and a payment equivalent to 3 years of basic salary, statutory compensation and participation in pension insurance was offered. The fourth option, for individuals under the age of 40, was to provide support for advanced studies, in the form of subsidies for tuition. There were 49,042 individuals who completed the competition process, of whom 20,831 were eventually re-employed.

Although the number of health staff, whether qualified or unqualified in the 15 sample counties decreased, the number of inpatient and outpatient visits and each member of staff’s workloads increased, meaning that their work efficiency improved (Table 2). In the interviews and seminars, all the administrators and frontline healthcare workers noted that the income of frontline healthcare workers increased after the reforms. However, the increased income did not fully reflect the increased workload. The frontline healthcare staff felt satisfied with the current status and the reform in their institution from the satisfaction survey results.

**Table 2:** Changes in human resource distribution in primary healthcare institutions after the reform

	**2008**	**2009**	**2010**
Total number of health personnel	16751	17324	13650
Total number of health technicians	12611	13109	11982
Proportion of health technicians (%)	75.29	75.67	87.78
Number of times of frontline healthcare worker’ daily workload	916.13	939.84	1035.55

Data were summarized from the statistical reports chosen.

3. **Compensation mechanism for primary healthcare institutions after the comprehensive reform**

The reforms successfully altered the main source of income for primary healthcare institutions in Anhui from drug sales to government compensation and charges for health services. Government compensation included specific payments for personnel expenses and operational costs, public health funding, balance of payments, grants for margin of expenses, and general income. Charges for health services consisted mainly of the general performance fee for each patient [10].

The results suggested an apparent change in the revenue structure. A gradual increase was seen, with the proportion of income from government increasing by 18.09% in 2009 and 41.09% in 2010. The proportion of income from drugs revenue decreased by 44.57% in 2009 and 25.19% in 2010 (Table 3).

**Table 3:** Changes in proportion of institutional revenue coming from different sources

	**2009**	**2010**
Proportion of income coming from government (%)	18.09	41.09
Proportion of income coming from drugs revenue (%)	44.57	25.19

Data were summarized from the statistical reports chosen. The government financial compensation did not include any funding through social medical insurance.

During the reforms, the number of patient visits for diagnosis and treatment increased. At the same time, there was also an increase in the total number of outpatient visits to primary healthcare institutions (Table 4).

**Table 4:** Changes in numbers of different visits in primary health care institutions

	**2008**	**2009**	**2010**	**% change (2009/2008)**	**% change (2010/2008)**
Diagnosis and treatment visits in primary health care institutions (ten thousand persons)	1155.33	1232.03	1240.79	6.64	7.35
Outpatient visits in primary health care institutions (ten thousand persons)	1106.2	1178.88	1199.25	6.57	8.41
Discharges in primary health care institutions (ten thousand persons)	49.13	53.16	41.54	8.20	âˆ’15.45

Data were summarized from the statistical reports chosen.

4. **Strengthening the primary healthcare system after the comprehensive reform**

Since the implementation of medical reform, Anhui has increased its fiscal input for the construction and transformation of primary health care institutions. After the reform, the number of primary healthcare institutions had increased, and the hardware subsequently improved. In 2008–2010, 1,195 standardized township hospitals, 14,134 village clinics, and 1,234 community health service institutions were constructed.

## Discussion

During the first round of comprehensive reforms of primary healthcare institutions in Anhui province, the visit conditions, personnel structure of medical staff, income structure, drug price, costs of treatment, and patterns of healthcare use by patients all changed. Among these, the changes in hardware facilities, personnel structure of medical staff, income structure, and drug price were a direct effect of reform. However, the treatment costs and patterns of healthcare use by patients could have been affected by multiple factors in addition to or instead of the reforms.

Eight hundred and fifty-seven drugs were approved for use in primary healthcare institutions. The overall quality of drugs procurement improved, with 34.6% of drugs coming from the top 100 medical enterprises, and 57.6% of drugs coming from the top 400 medical enterprises. Purchase price fell by 10% compared with the previous average price of drugs purchased by primary healthcare institutions [11]. However, the prescribing behavior of doctors did not change, with the use rate of antibiotics falling by just 0.62%, which was not a statistically significant difference. It is possible that changes in ways of working in primary healthcare institutions would take longer to be reflected in the prescription behavior of doctors, and that a change will be seen at a later stage. The total cost of treatment per outpatient visit decreased from 35.29 RMB to 31.64 RMB although the average inpatient visit cost increased from 799.05 RMB to 992.60 RMB. The decrease in outpatient cost might be related to the decreases in drug prices. However, unlike outpatient costs, the decrease in drugs cost therefore did not reduce the costs of inpatient treatment. An apparent change was found in the revenue structure. A gradual increase was seen, with the proportion of income from government increasing by 18.09% in 2009 and 41.09% in 2010. The proportion of income from drugs revenue decreased by 44.57% in 2009 and 25.19% in 2010. Therefore, it was suggested that the increased inpatient cost may due to have higher examination, treatment, and other costs in addition to the drugs cost from inpatient treatments. In total, the decrease seen in treatment costs could largely be attributed to the decrease in drugs prices.

Professional and technical positions accounted for 87.7% of the total staff after the reforms, an increase of 12.5% over the position before reform. Among these staff, 94.2% had a college degree or above after reform, 16.3% higher than before. 82.8% had junior titles or above, an increase of nearly 11% over the previous position, and the proportion of people with practitioner (assistant) qualifications, such as doctors, increased by nearly 10% over the previous 36% [12]. After reform, the workload of frontline healthcare workers increased, and their work efficiency improved. However, analysis of the costs of treatment indicated that the decrease in cost was mainly due to the decrease in the prices of drugs, and that the overall quantity of drugs prescribed had not decreased. It was also clear from analysis of patient behavior that patients with simple conditions tended to go to primary healthcare institutions, while those with more complex conditions preferred non-primary healthcare institutions. The reforms have therefore made the entire county clinic structure more workable.

Between 2008 and 2010, both the total number of diagnosis and treatment visits in primary health care institutions and the total number of outpatient visits to primary healthcare institutions increased. The percentage change in the number of diagnosis and treatment visits in primary health care institutions and the total number of outpatient visits to primary healthcare institutions was substantially larger. However, the discharges in primary health institutions did not change too much. This finding indicated that the major functional orientation of primary healthcare institutions was changed, so that they now mainly undertake basic medical services, especially in areas with low economic activity. The changes seen were therefore consistent with the reforms’ objectives.

Although the comprehensive reform in Anhui had some positive effects, some problems emerged. The reform changed the compensation system for medical institutions and the income distribution mechanism for medical personnel, but lacked a rigid evaluation system for those areas of income that did not fall into either category. In interviews and seminars, administrators and frontline healthcare workers in primary healthcare institutions noted that the income of frontline healthcare workers increased after the reforms. However, the increased income did not fully reflect the increased workload, and those who worked the hardest did not necessarily get paid the most. This led to some medical staff becoming demotivated. In the next step of reform, performance evaluation will need to be strengthened further, and the proportion of pay that is performance-based should be increased. Moreover, information technology and other means should be used to ensure the accuracy and fairness of performance evaluation. The income gap between medical staff should be widened appropriately to fully embody the principle of more pay for more work. A number of doctors and patients also observed that there were shortages of drugs. This was caused by a number of factors. First, different doctors had different practices relating to drug use, which could not be changed within such a short period to reflect agreed supply levels. Second, the overall quality and education level of health staff in primary healthcare institutions was relatively low. Some may not pass the formal examination and even lacked knowledge about medication. Third, a single supply source was chosen for each drug through the centralized bidding process. Fourth, secondary and tertiary hospitals were unaffected by the reforms, causing an inconsistency in medicine use in secondary and tertiary hospitals compared with primary care. A large proportion of patients in primary healthcare have been referred by higher-level hospitals, but the prescription drugs used in those hospitals are not offered in primary care. To address this problem, the essential medicine list should be adjusted to ensure that medicines are available if they are considered necessary for prevention and treatment, clinically preferred, high quality, reasonably priced, and linked to medicines used by higher-level hospitals. To a certain extent, this adjustment could ease contradictions in drug use.

## Conclusion

The comprehensive reform of primary healthcare institutions changed the structure of medical personnel, increased efficiency, optimized the diagnosis and treatment structure, and reduced drugs prices to some extent. However, the decrease seen in treatment costs could largely be attributed to the decline in drugs prices. The increase in diagnosis and treatment visits to primary healthcare institutions was not directly related to the reforms. The behavior of medical personnel did not change significantly. At the same time, two problems emerged from the reforms. First, the enthusiasm of medical staff decreased, and second, the supply of drugs could not adequately meet the demand. Approaches such as strengthening performance evaluation, further mobilizing frontline healthcare workers, improving training on drug use, and educating patients should be undertaken in the future.

### Limitations

This study has three main limitations. First, there may be some problems with data analysis because some information was unavailable. Second, the analysis was undertaken very soon after initial implementation of the reforms so that effects and problems may not yet have emerged or be fully apparent. The study would need to be repeated at a later stage to overcome this limitation. Third, the analysis for urban and rural settings was not separated in this study, and there might be significant differences between these settings; further analysis would be necessary to resolve this question.

## Competing interests

All authors declare that they have no competing interests.

## Authors’ contributions

QL designed the prescription study, collected the data, conducted the data analysis and prepared the manuscript; XT contributed to the design and analysis of the study and the preparation of the manuscript; JT have participated in acquisition of data; and XZ assisted with the data analysis and reviewed the manuscript. All authors read and approved the final manuscript.

## Pre-publication history

The pre-publication history for this paper can be accessed here:

http://www.biomedcentral.com/1472-6963/14/268/prepub
